# Acute and Subchronic Toxic Effects of the Fruits of *Physalis peruviana* L.

**DOI:** 10.1155/2013/707285

**Published:** 2013-11-28

**Authors:** Basak Ozlem Perk, Sinem Ilgin, Ozlem Atli, Hale Gamze Duymus, Basar Sirmagul

**Affiliations:** ^1^Department of Pharmaceutical Toxicology, Faculty of Pharmacy, Anadolu University, 26470 Eskisehir, Turkey; ^2^Department of Pharmacognosy, Faculty of Pharmacy, Anadolu University, 26470 Eskisehir, Turkey; ^3^Department of Pharmacology, Faculty of Medicine, Osmangazi University, 26480 Eskisehir, Turkey

## Abstract

The fruit of *Physalis peruviana* L. (PPL) has been traditionally used as antispasmodic, diuretic, antiseptic, sedative, and analgesic all over the world. We aimed to perform qualitative content analysis of the fruits of PPL and to clarify the *in vitro* genotoxicity and *in vivo* acute and subchronic toxicity of the fruit. Lyophilized fruit juice does not induce genetic damage. In the acute toxicity studies, LD50 value of the fruit was found to be more than 5000 mg kg^−1^ for both sexes. According to the subchronic toxicity studies, hepatic, renal, and hematological toxic effects were not induced in both sexes. Plasma troponin I (only in the group treated with 5000 mg kg^−1^ of lyophilized fruit juice) and troponin T levels were significantly increased in male groups treated with lyophilized fruit juice compared to the control group. Furthermore, potassium level was significantly increased in the male group treated with 5000 mg kg^−1^ of lyophilized fruit juice. These findings were considered to indicate the myocardial damage particularly in the male group treated with 5000 mg kg^−1^ of lyophilized fruit juice. In conclusion, lyophilized fruit juice of PPL is shown to induce cardiac toxicity only at high doses and in male gender.

## 1. Introduction

Herbs and herbal preparations are in use for the treatment of various diseases throughout human history. According to the World Health Organization (WHO) statistics, 70–80% of the world's population appeals to plant-derived traditional treatment methods for the solution of health problems [1, 2]. However, it is well known that consumption of plants and plant products, of which the content and the toxicity profile and safe dose were not determined, by humans and animals may cause severe toxicity problems [2].

The fruits of *Physalis peruviana *L. (*Solanaceae*) are also named as goldenberry, gooseberry, cape gooseberry, and wintercherry fruits all over the world [3, 4]. *Physalis* species are grown naturally and cultured in a wide range of countries including North and South African countries, India, Australia, New Zealand, Ecuador, Venezuela, Colombia, Chile, and Peru [4]. In Turkey, *Physalis pubescens *L. is grown in Artvin region, *Physalis alkekengi *L. is grown in the regions of Kutahya, Antalya, Bitlis, Istanbul, Sakarya, Samsun, and Karaman, and the fruit of *Physalis peruviana* L. is cultured in Antalya [5]. The fruit contains polyunsaturated fatty acids, carbohydrates, A, B, C, E, and K1 vitamins, phytosterols, essential minerals (phosphorus, iron, potassium, and zinc), physalin, and withanolides [4, 6–9].

The fruit of *Physalis peruviana* L. is used among people as antispasmodic, diuretic, antiseptic, sedative, and analgesic as well as in the treatment of throat infections and elimination of intestinal parasites and amoeba and to strengthen the optic nerve [10], but, in Turkey, fresh or dried fruits and preparations containing fruit extract of *Physalis peruviana* L. are frequently used, in the aim of weight loss in recent years. Case studies showed that fruits might cause side effects such as hypertension, ventricular tachycardia, and manic episode in our country [11–13]. Furthermore, biological activity assay carried out on the fruit, stem, and leaves of the plant has demonstrated that it has hepatoprotective and antioxidant effects as well as cytotoxic effects on certain cancer cell lines [4, 14–19].

It is interesting that the toxicity studies on the fruits of *Physalis peruviana *L.—a widely used plant in different geographical regions all over the world—are lacking. Our study aimed to determine the possible acute toxic effects of the fruit by using *in vivo* animal models and subchronic toxicity at the organ level as well as to assess the *in vivo* genotoxicity of the fruits of *Physalis peruviana* L.

## 2. Materials and Methods

### 2.1. Materials

Cultured fruits were obtained from Antalya, Turkey. The fresh fruits were separated from their calyxes and they were cut into small pieces. These pieces were homogenized. After homogenization, the pulp was filtered in the cold. Meanwhile, the unsoluble particles were seperated from the juice. The freeze-dried fruit juice was kept in a well-closed amber-colored flacon until performing the assays. During this study, totally 20 Kg fresh fruits were prepared for the experiments and 3.9 Kg extract was obtained. The extraction yield was calculated as 19.5% of the fresh fruits.

The chemicals used were obtained from the following sources: ketamine (Ketalar) (Phizer, Turkey); xylazine (Bayer, Turkey). For the measurements of lactate dehydrogenate (LDH) (BioAssay Systems, CA, USA), creatinine kinase-MB (CK-MB) (BioCheck, CA, USA), troponin I (TI) (BioCheck, CA, USA) and troponin T (TT) (Cusabio, China) levels ELISA kits were used according to the manufacturer's instructions in plasma samples. For the measurements of potassium and sodium levels ELISA kits from Diazyme Laboratories, CA, USA, were used. Blood aspartate amino transferase (AST), alanine amino transferase (ALT), alkaline phosphatase (AP), total and direct bilirubin, albumin, total protein, urea, creatinine, and glucose levels were determined by colorimetric kits from Biolabo SA (Châtel-St-Denis, Switzerland). Genotoxicity was evaluated by umuC assay using umuC Easy CS Kit (Xenometrix AG, Gewerbertrasse, Switzerland).

### 2.2. Phytochemical Screening

Phytochemical tests were carried out using lyophilized fruit juice of *Physalis peruviana* L. to identify the constituents by using standard colorimetric methods: carbohydrates (*Fehling's* and *Molisch's* reactions), tannins (*Ferric chloride* reaction), basic alkaloids (*Mayer's*, *Dragendorff *and *Hager's* reactions), tropane alkaloids (*Vitali Morin* reaction), polyuronides (precipitation with acetone), proteins (Biuret and Ninhydrin's assays), carotenoids (*Carr Price's* reaction), fixed oil (*Sudan red* reaction), saponins (*Salkowski* and *Lieberman-Burchard's* reactions), cyanogenetic glucoside (*Sodium picrate* assay), flavon glucoside (*Shibata's* reaction), and cardiotonic glucoside (*Baljet* and *Kedde's* reactions) [20–23].

### 2.3. umuC Assay

*Salmonella typhimurium* strain TA1535/pSK1002 was used for the assay. TG medium (200 *μ*L) was added to the vial to obtain homogenous suspensions of *Salmonella* strain. 10 *μ*L of ampicillin (50 mg/mL) was added to 10 mL TG medium (=TGA medium) in 50 mL culture tubes. 50 *μ*L of *Salmonella* suspension was mixed with 10 mL TGA medium. Negative control was devoid of *Salmonella* TA1535/pSK1002. The culture tubes were loosely capped, to allow aeration, and incubated in a shaker (SI-600, Jeio Tech, Korea) at 37°C, 250 rpm for 14–16 h. The “overnight grown” cultures were diluted 10 times with TG medium and the absorbance was measured at 600 nm. Positive controls were prepared with S9 (2-aminoanthracene) and without S9 (4-nitroquinoline). TGA medium and S9 fraction were added to each well of plate. Then, lyophilized fruit juice of PPL was added to wells at 5, 2.5, 1.25, and 0.63 mg/mL concentrations. The plates were incubated at 37°C, 120–150 rpm for 2 hours. During the 2 hours, a second plate was prepared with TG medium with freshly added ampicillin to all wells (for 1 plate: 28 *μ*L ampicillin stock (50 mg/mL) to 28 mL TG medium). After 2 hours, 30 *μ*L of the contents of the first plate was transferred to the second plate. OD600 of the second plate was read. Then, the second plate was incubated at 37°C, 120–150 rpm for 2 hours. During the 2 hours, a third plate was prepared with 150 *μ*L B-buffer/ONPG (o-nitrophenyl-*β*-D-galactopyranoside) mixture (for 1 plate: 15 mL of B buffer, 40.5 *μ*L 2-mercaptoethanol, and 1 mL ONPG solution) and prewarmed to 28°C. At the end of the 2 hours of incubation, the second plate was mixed and OD600 of the plate was read. Then, 30 *μ*L of each well of the second plate was transferred to the third plate. The third plate was incubated at 28°C, 120–150 rpm for 30 minutes. After 30 minutes, 120 *μ*L of stop reagent was added to each well. The plate was mixed and OD420 of the plate was read. The results were determined with umuC Easy CS Excel Programme.

### 2.4. Experimental Design

#### 2.4.1. Animals

Female-male Wistar rats weighing 200–250 g were obtained from our own animal facility. Rats were housed under controlled temperature (22°C) and lighting (12/12-hour light dark cycle) with free access to food and water. The experimental protocol was approved by the Local Ethical Committee on Animal Experimentation of Anadolu University, Eskisehir, Turkey.

#### 2.4.2. Acute Oral Toxicity Study

Acute toxicity tests were performed in accordance with the 2008 regulations of Organization for Economic Cooperation and Development (OECD Test Guideline: 423). Lyophilized fruit juice of PPL was dissolved in distilled water and orally administered to 5 male and 5 female rats at a dose of 5000 mg kg^−1^. The animals were monitored during a 24-hour period for clinical symptoms (aggression, sedation, somnolence, tremor, catatonia, paralysis, convulsions, changes in skin, eyes and mucosal membranes, asphyxia, salivation, and diarrhea). Following the 24 hours, number of dead animals from each group was recorded. Because there were no dead male or female rats at the end of this period, they were subjected to a 14-day observation period. After this period, the rats were killed by exposure to high dose of ether anesthesia and liver; kidney, heart, and lung tissues of the animals were evaluated macroscopically.

#### 2.4.3. Subchronic Oral Toxicity Study

Subchronic toxicity tests were performed in accordance with the 1998 regulations of Organization for Economic Cooperation and Development (OECD Test Guideline: 408). Lyophilized fruit juice of PPL diluted with distilled water was orally administered to 10 male and 10 female rats for a period of 90 days at the doses of 100, 1000, and 5000 mg kg^−1^ day^−1^ in a volume of 1 mL/100 g. Control group (*n* = 10) received an equal volume of distilled water for the same period of time. Toxicity symptoms and mortality were recorded on a daily basis and body weights of the animals were measured weekly during this time period. At the end of 90 days, the animals were anesthetized by intraperitoneal injection of 60 mg kg^−1^ ketamine and 5 mg kg^−1^ xylazine. Blood samples for haematological and biochemical analyses were collected by withdrawing the blood from the right ventricle of the anesthetized animals by using a syringe. The animals were killed by withdrawing large amounts of blood from the heart and heart, liver, kidney, lung, spleen, and ovary or testicular tissue were removed and weighed.

### 2.5. Haematology and Serum Biochemistry

Hematologic toxicity was evaluated by determining the white blood cell count, the percentages of neutrophils, lymphocytes, monocytes, eosinophils, basophils, and red blood cells, hematocrit, red blood cell volume, mean corpuscular hemoglobin, mean corpuscular hemoglobin concentration, platelet count, and mean platelet volume on Coulter LH 750 Hematology Analyzer device (BeckmanCoulter, Inc) in the Hematology Laboratory of Eskisehir Osmangazi University Faculty of Medicine, Turkey.

Biochemical parameters used for the evaluation of toxic effects at the organ level were determined in plasma samples by using commercially available kits. In accordance with the manufacturer's instructions, cardiac toxicity was evaluated by measuring LDH, CK-MB, TI, and TT levels, hepatic toxicity was evaluated by measuring AST, ALT, AP, total and direct bilirubin, albumin, and total protein levels, renal toxicity was evaluated by measuring urea, creatinine, potassium, and sodium levels, and metabolic toxicity was evaluated by measuring blood glucose level.

### 2.6. Statistics

The percent increase in body weights of animals was calculated and repeated measurements were performed by two-way analysis of variance (ANOVA two-way repeated measures) and multiple comparisons by Holm-Sidak post-hoc test with using SigmaStat 3.5 program. The *P* values less than 0.05 were considered as significant. The statistical analysis for all other parameters was performed on SigmaStat 3.5 program by using Kruskal-Wallis one-way analysis of variance (one-way ANOVA) and Tukey's post-hoc test for multiple comparisons. All comparisons were considered as significant at a *P* level of <0.05.

## 3. Results

### 3.1. The Content of Lyophilized *Physalis peruviana* L. Fruit Juice

The qualitative analysis revealed that the lyophilized fruit juice of PPL contains carbohydrate, protein, fixed oils, alkaloids, polyuronides, fruit juice of PPL died during, flavone glycosides, and cardiotonic glycosides.

### 3.2. Genotoxicity Study

Administration of different concentrations of lyophilized fruit juice of PPL resulted in an induction ratio of less than 1.5 and the growth factor level of more than 0.5 in the presence and absence of enzyme fraction with the *Salmonella typhimurium* TA1535 [pSK1002] strain. This may imply that administration of lyophilized fruit juice of PPL does not induce any genetic damage.

### 3.3. In-Life Parameters

#### 3.3.1. Acute Oral Toxicity Study

No death was observed 24 hours after the oral administration of 5000 mg kg^−1^ lyophilized fruit juice of PPL to the groups of animals consisting of 5 male and 5 female rats. No behavioral symptoms such as aggression, sedation, and drowsiness, no neurological symptoms such as tremor, catatonia, paralysis, and convulsion, no changes in skin, eyes, and mucosal membranes, and no asphyxia, salivation, and diarrhea were observed during this 24-hour period and during 14-day observation period. At the end of observation period, no macroscopic necrotic focus was observed in the heart, liver, kidney, lung, stomach, spleen, and ovary or testicular tissues of the animals.

#### 3.3.2. Subchronic Oral Toxicity Study

It was an important finding that 50% of male animals treated with 5000 mg kg^−1^ day^−1^ lyophilized fruit juice of PPL died during the 90-day treatment period.

**Figure 1:**
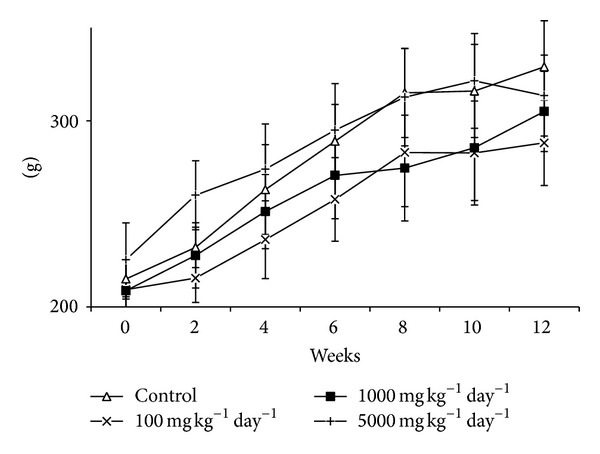
Mean body weight in male rats treated with lyophilized fruit juice of PPL for subchronic period.

When the percent body weight changes in the male animals were compared according to the doses and weeks, there was a significant decrease in percent body weight in the group treated with 5000 mg kg^−1^ day^−1^ of lyophilized fruit juice of PPL compared to the control group at week 12. No significant difference was found between the control group and the male animals treated with 100 or 1000 mg kg^−1^ day^−1^ of lyophilized fruit juice of PPL (Figure 1).

**Figure 2:**
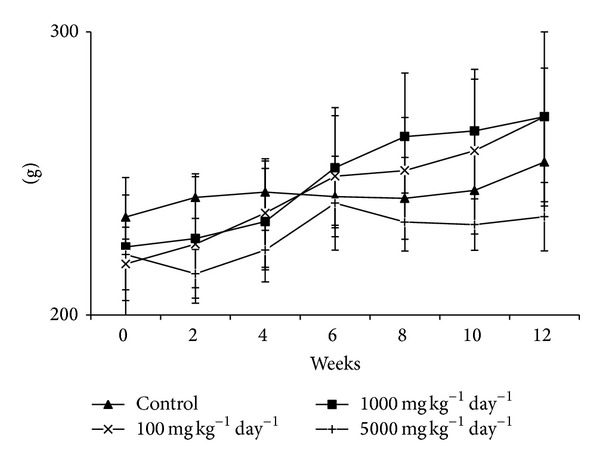
Mean body weight in female rats treated with lyophilized fruit juice of PPL for subchronic period.

When the percent body weight changes in female animals were compared according to the doses and weeks, there was a significant increase in percent body weight in the groups treated with 100 and 1000 mg kg^−1^ day^−1^ of lyophilized fruit juice of PPL compared to the control groups at weeks 8, 10, and 12. No significant difference was found between the control group and the female rats treated with 5000 mg kg^−1^ day^−1^ of lyophilized fruit juice of PPL (Figure 2).

### 3.4. Organ Weights

The percent organ weight/body weight ratios calculated for male and female animals treated with 100, 1000, and 5000 mg kg^−1^ day^−1^ lyophilized fruit juice of PPL were not significantly different from the control group. Similarly, there was no difference when PPL-treatment groups were compared with each other (Tables 1 and 2).

### 3.5. Hematology/Plasma Biochemistry

Hematological biomarkers were not significantly different in male and female groups treated with 100, 1000, and 5000 mg kg^−1^ day^−1^ lyophilized fruit juice of PPL for 90 days compared to the control groups. Similarly, there was no statistically significant difference between the PPL-treatment groups. In contrast, female rats treated with 5000 mg kg^−1^ day^−1^ lyophilized fruit juice of PPL had significantly higher blood platelet levels compared to the controls. Hematological parameters in the groups are shown in Tables 3 and 4.

Plasma hepatic and renal biomarkers were not significantly different in male and female groups treated with lyophilized fruit juice of PPL compared to the control groups. Similarly, there was no statistically significant difference between the PPL-treatment groups. It was noteworthy that serum potassium level was significantly higher in male animals treated with 5000 mg kg^−1^ day^−1^ lyophilized fruit juice of PPL compared to the controls (Tables 5 and 6).

Plasma glucose level did not differ in female treatment group compared to the control group. In contrast, male animals treated with lyophilized fruit juice of PPL had significantly higher plasma glucose levels compared to the controls (Tables 5 and 6).

Although plasma CK-MB, LDH, and troponin T levels were not significantly different in female treatment groups, plasma troponin I level was found to be significantly increased in female animals treated with 100 mg kg^−1^ day^−1^ lyophilized fruit juice of PPL compared to that in the controls. On the other hand, plasma CK-MB and LDH levels were not significantly different in male treatment groups; troponin I level was significantly increased in only male animals treated with 5000 mg kg^−1^ day^−1^ lyophilized fruit juice of PPL and troponin T level was significantly increased in male rats treated with 100, 1000, and 5000 mg kg^−1^ day^−1^ lyophilized fruit juice of PPL compared to those in the controls (Tables 5 and 6).

## 4. Discussion

In recent years, there is an increasing emphasis on the safety studies for plants and plant products, and effectiveness and safety of the plants and plant products are established through scientific research. The complexity of herbal preparations and their natural biologic variations makes it necessary to establish their safety, effectiveness, and quality [24, 25].

Like the widely used Ames test, Umu-C test is also a comparable method that shows high correlation with rodent mutagenicity [26, 27]. The results of the Umu-C test providing rapid prediction for determining the genotoxicity suggest that lyophilized fruit juice of PPL has no genotoxic effects in the presence or absence of S9 enzyme fraction.

Although the literature is lacking about the LD50 value of the substance, no death was observed in the animals 24 hours after the administration of 5000 mg kg^−1^ day^−1^ lyophilized fruit juice of PPL, indicating that the LD50 value of the substance is more than 5000 mg kg^−1^.

The increases or decreases in body weight usually reflect physiological changes such as changes in liver function, hormonal changes, and poor absorption of protein and amino acids. Because the expected weight gain was not observed in the female control group, the decreased body weight found in the groups treated with high dose of lyophilized fruit juice of PPL was not considered as the reflection of toxicological effect of the fruit. Instead, the stress induced by the gavage and the inflammation induced in the digestive tract by gavage tube in both groups might lead to difficulty in eating in the animals. On the other hand, the percent body weight changes did not differ significantly between the male animals from control, low-dose, and intermediate-dose groups, while they were significantly decreased in the last week in male animals treated with high-dose of lyophilized fruit juice of PPL compared to the control animals. This can be as a dose- and time-dependent toxic effect. However, because this effect was not evident in female animals treated with high-dose of lyophilized fruit juice of PPL, the lyophilized fruit juice of PPL may not have a negative effect on the weight gain or male animals may be more sensitive to this effect. On another hand, the diarrhea observed in both male and female animals, particularly in those treated with 5000 mg kg^−1^ day^−1^ lyophilized fruit juice of PPL, can be associated with the weight loss found in these groups. In our study, qualitative analysis demonstrated that the lyophilized fruit juice of PPL contains mucilage. The weight loss found in the male animals treated with high dose of lyophilized fruit juice of PPL can be associated with the diarrhea due to the mucilage content [28, 29]. Sugar derivatives like sorbitol, mannitol, and lactulose are present in fruits and they act as osmotic laxatives [30]. The diarrhea observed in both male and female animals, particularly in those treated with 5000 mg kg^−1^ day^−1^, may be identified with these sugar derivatives in addition to the mucilage content of lyophilized fruit juice of PPL. However, at this point, it is a great limitation for the interpretation of this process that the food consumption of the animals was not recorded in this study. Moreover, because there was no significant difference in relative organ weights in the animals groups from both sexes compared to the controls, it can be considered that the lyophilized fruit juice of PPL did not trigger atrophic and hypertrophic processes at the organ level.

The changes in the hematological parameters found in animal studies provide prediction of toxicity on hematological system in humans [31]. In this study, the hematological parameters did not differ significantly between both treatment groups and control groups except for platelet count. Platelet count was higher for animal from both sexes and this difference was statistically significant for female animals treated with high dose of lyophilized fruit juice of PPL, suggesting that the lyophilized fruit juice of PPL may induce thrombocythemia, particularly in female animals. The etiologic causes of primary thrombocythemia not accompanying any disease are not known. Thrombocythemia may develop due to the anemia, cancer, or infectious diseases and may cause serious complications such as blood clots, bleeding, myocardial infarction, and stroke [32]. The results of other hematological parameters measured in this study can imply that the lyophilized fruit juice of PPL does not have toxic effects on hematopoiesis and the percentage of blood cell counts.

Increased plasma ALT and AST levels are associated with hepatic damage or the changes in the permeability of hepatocyte membrane [33, 34]. Because AST is widely distributed in the body including primarily the liver, muscles, and red blood cells, ALT is considered as a more specific enzyme for liver function. Plasma ALP level increases in the case of obstruction of bile flow [35]. Furthermore, decreased level of plasma proteins reflects chronic liver injury. Plasma albumin levels are used to evaluate the synthesis function of the liver [36, 37]. In addition, increased bilirubin levels confirm the disturbances in the liver function [34, 35]. On the other hand, total protein level decreases in the case of protein loss due to poor nutrition, malabsorption, and kidney or liver damage [37]. The levels of hepatic biomarkers and the relative liver weight were not different in the animals from both sexes compared to the controls, suggesting that the lyophilized fruit juice of PPL does not induce hepatotoxicity.

Serum creatine, urea, sodium, and potassium levels are used in the assessment of renal toxicity [34, 36, 38]. Plasma electrolytes, creatinine, and urea levels measured in this study to assess the renal toxicity did not significantly differ in male and female animals treated with lyophilized fruit juice of PPL compared to the controls. The increased serum potassium level found in male animals treated with 5000 mg kg^−1^ day^−1^ lyophilized fruit juice of PPL compared to the controls was not considered as an indicator of renal toxicity, because other biomarkers of renal toxicity did not significantly differ between the treatment groups and the control group. The levels of renal biomarkers and the relative renal weight were not different in the animals from both sexes compared to the controls, suggesting that the lyophilized fruit juice of PPL does not induce renal toxicity.

Carbohydrate metabolism is assessed by measuring the blood glucose levels, with high levels being considered as the herald of diabetes mellitus [37]. Animals from both the control groups and treatment groups had normal or near-normal (50–135 mg/dL) blood glucose levels. Minimal increases found in the blood glucose level in animals treated with lyophilized fruit juice of PPL might be resulted from the high carbohydrate content of the fruit of *Physalis peruviana* L.

The increased blood levels of cardiac biomarkers are considered as indicators of the myocardial damage. The levels of LDH, total CK, AST, and troponins are used for the assessment of this damage. However, the measurement of AST, CK, and LDH activities has a poor sensitivity and specificity for the myocardial tissue and therefore their use in clinical practice is limited [39–41]. Furthermore, because the half-life of CK-MB (0.5–1 hours in rats) and LDH (0.5–6 hours in rats by the type of isoenzymes) are relatively short, they are commonly used in determining acute skeletal injuries and myocardial damage [41–43]. However, because LDH and CK-MB are not specific to the cardiac tissue, they lost their significance as the cardiac biomarkers and now troponins are used instead of them [44]. Troponin levels being the gold standard in determining the cardiac ischemic injury were significantly higher in male animals, particularly in those treated with 5000 mg kg^−1^ day^−1^ lyophilized fruit juice of PPL, suggesting that the cardiac toxicity may be induced by lyophilized fruit juice of PPL. Because of the long half-lives of troponins, it is possible to determine the changes in these markers even days after ischemic injury. Most probably, the CK-MB and LDH levels were also increased in the acute phase. However, these markers have limited use to show long-term effects because of their short half-lives. Another finding confirming the cardiac toxicity is the increased serum potassium level particularly in male animals treated with high dose lyophilized fruit juice of PPL. Myocardial injury resulting from ischemia-reperfusion damage might increase the serum level of this intracellular cation. Increased serum potassium level has been suggested to indicate the myocardial injury during the early phase of ischemia-reperfusion [39]. However, because blood samples were not obtained from dead animals, biochemical analyses for acute causes could not be performed and possible myocardial infarction could not be demonstrated. It is noteworthy that cardiac troponin and serum potassium levels were not significantly different in female animals treated with lyophilized fruit juice of PPL compared to the controls, suggesting that the lyophilized fruit juice of PPL did not induce cardiac toxicity in female animals.

The results of this study on cardiac biomarkers suggest that while lyophilized fruit juice of PPL induces cardiotoxicity in male animals, it is not true for female animals. This difference between the sexes may be associated with the level of estrogen hormone in females. It is well known that prevalence of cardiovascular disease is lower in premenopausal women than men [45–48]. Previous studies have shown a protective effect of estrogen against the development of cardiovascular disease [49, 50].

The qualitative analysis provides a general opinion about the content of lyophilized fruit juice of PPL. Although alkaloid derivative compounds were found in diagnostic reactions, no tropane alkaloids were detected. Furthermore, the diagnostic reactions specific for cardiotonic glycosides and saponins were also positive. Cardiotonic glycosides are directly effective on heart muscle. These glycosides increase the heart muscle contraction force and are used in the treatment of patients with congestive heart failure. However, these agents have a narrow therapeutic index and their effect is dose dependent. The cardiac glycosides isolated from *Digitalis purpurea* (Scrophulariaceae) include digitoxin, digitonin, and digitaline. High doses of glycosides increase the vagal tonus in atrioventricular and sinoatrial nodes by affecting the electrical conduction in the heart, leading to cardiac arrhythmias and myocardial infarction. The cardiac symptoms induced by these glycosides include increased strength of cardiac contractions, prolongation of the diastole, abnormalities in heart rate and cardiac rhythm, hyperkalemia, and ventricular tachycardia [51]. It can be suggested that the fruits of *Physalis peruviana* L. may have a cardiac toxicity profile similar to that of digitalis.

The results of the present study suggest that the lyophilized fruit juice of PPL may not induce genotoxicity and may not lead to hematological, hepatic, and/or renal toxicity in both sexes. It was found that repeated high doses of lyophilized fruit juice of PPL may cause cardiotoxicity in male, particularly predisposed individuals. Also, in a case study of Turkey, subchronic use of dried fruits of PPL for weight-loss in an adult male was shown to be a possible cause of subarachnoid hemorrhage related to hypertension [11]. It is obvious that target organ-specific studies are needed at this point. Future studies will aim to elicit the predicted cardiac toxicity profile of the lyophilized fruit juice of PPL and the active biologically compound(s) responsible for these effects by narrowing the dose range, using telemetric systems to obtain routine ECG records and performing regular blood pressure measurements.

## Figures and Tables

**Table 1:** Effect on organ weights% of male rats treated with lyophilized fruit juice of PPL for subchronic period.

	Male			
	Control	100 mg kg^−1^	1000 mg kg^−1^	5000 mg kg^−1^
Lungs (%)	0.58 ± 0.24	0.75 ± 0.29	0.63 ± 0.18	0.62 ± 0.17
Liver (%)	3.83 ± 1.13	3.82 ± 1.32	3.52 ± 0.29	3.78 ± 1.04
Heart (%)	0.45 ± 0.05	0.45 ± 0.10	0.40 ± 0.05	0.44 ± 0.03
Kidneys (%)	0.72 ± 0.14	0.70 ± 0.14	0.67 ± 0.18	0.79 ± 0.14
Spleen (%)	0.47 ± 0.15	0.57 ± 0.13	0.42 ± 0.08	0.56 ± 0.08
Testes (%)	1.14 ± 0.15	1.24 ± 0.13	1.26 ± 0.08	1.27 ± 0.09

(% organ weights/body weights (g g^−1^)) (*n*: 7).

**Table 2:** Effect on organ weights% of female rats treated with lyophilized fruit juice of PPL for subchronic period.

	Female			
	Control	100 mg kg^−1^	1000 mg kg^−1^	5000 mg kg^−1^
Lungs (%)	0.46 ± 0.34	0.54 ± 0.11	0.56 ± 0.35	0.59 ± 0.33
Liver (%)	3.06 ± 0.32	3.52 ± 0.31	3.04 ± 1.18	3.34 ± 0.60
Heart (%)	0.45 ± 0.04	0.43 ± 0.03	0.42 ± 0.06	0.46 ± 0.04
Kidneys (%)	0.69 ± 0.06	0.66 ± 0.07	0.65 ± 0.14	0.73 ± 0.16
Spleen (%)	0.45 ± 0.08	0.56 ± 0.15	0.52 ± 0.06	0.54 ± 0.08
Ovaries (%)	0.08 ± 0.01	0.12 ± 0.01	0.10 ± 0.01	0.10 ± 0.02

(% organ weights/body weights (g g^−1^)) (*n*: 7).

**Table 3:** Effect on hematological parameters after oral administration of lyophilized fruit juice of *Physalis  peruviana* L. during subchronic period in male Wistar rats (*n*: 7).

	Male			
	Control	100 mg kg^−1^	1000 mg kg^−1^	5000 mg kg^−1^
WBC (10^3^ *µ*L^−1^)	5.54 ± 0.93	5.68 ± 1.28	4.45 ± 0.69	5.30 ± 1.89
Neutrophils (%)	3.27 ± 2.12	4.45 ± 1.54	5.68 ± 1.64	6.08 ± 2.53
Lymphocytes (%)	90.83 ± 9.72	89.53 ± 5.27	85.65 ± 5.33	88.30 ± 4.08
Monocyte (%)	3.63 ± 3.98	3.72 ± 2.20	4.44 ± 2.45	2.39 ± 1.63
Eosinophils (%)	1.54 ± 1.97	1.76 ± 1.42	2.47 ± 1.60	2.59 ± 0.92
Basophils (%)	0.93 ± 0.28	2.27 ± 1.57	0.17 ± 0.10	0.40 ± 0.16
RBC (10^6^ *µ*L)	8.14 ± 0.41	7.98 ± 0.56	8.18 ± 0.39	8.03 ± 0.46
HGB (g dL^−1^)	15.06 ± 0.50	14.70 ± 1.08	15.40 ± 0.89	14.70 ± 0.53
Haematocrit (%)	43.00 ± 1.41	40.90 ± 3.16	42.77 ± 2.36	47.18 ± 4.09
MCV (fL)	52.89 ± 1.61	51.22 ± 1.60	51.30 ± 1.77	57.39 ± 4.49
MCH (pg)	18.53 ± 0.45	18.38 ± 0.46	18.44 ± 0.40	18.03 ± 0.61
MCHC (g dL^−^)	35.01 ± 0.47	35.92 ± 0.30	36.00 ± 0.55	31.57 ± 3.31
RDW-CV (%)	15.03 ± 1.13	16.84 ± 2.21	17.09 ± 1.27	19.40 ± 2.97
Platelet (10^3^ *µ*L^−^)	717.13 ± 92.93	795.00 ± 75.24	768.33 ± 34.42	918.00 ± 166.35
MPV (fL)	7.49 ± 0.38	7.12 ± 0.40	6.93 ± 0.16	7.30 ± 0.10

WBC: white blood cell; RBC: red blood cell; HGB: haemoglobin; MCV: mean corpuscular volume; MCH: mean corpuscular Hb; MCHC: mean corpuscular Hb conc; RDW-CV: coefficient variation of red cell distribution width; MPV: mean platelet volume.

**Table 4:** Effect on hematological parameters after oral administration of lyophilized fruit juice of *Physalis peruviana *L. during subchronic period in female Wistar rats (*n*: 7).

	Female			
	Control	100 mg kg^−1^	1000 mg kg^−1^	5000 mg kg^−1^
WBC (10^3^ *µ*L^−1^)	5.54 ± 0.93 2.38 ± 0.60	3.87 ± 1.22	2.60 ± 1.18	2.31 ± 0.98
Neutrophils (%)	7.32 ± 1.66	5.37 ± 1.48	8.88 ± 3.01	9.18 ± 3.80
Lymphocytes (%)	90.46 ± 2.80	90.72 ± 9.39	85.98 ± 6.16	87.86 ± 5.22
Monocyte (%)	2.10 ± 1.08	2.30 ± 1.51	2.49 ± 1.25	3.10 ± 2.08
Eosinophils (%)	0.09 ± 0.05	0.77 ± 0.58	1.48 ± 0.94	1.09 ± 0.75
Basophils (%)	0.37 ± 0.18	0.27 ± 0.13	0.34 ± 0.35	0.21 ± 0.10
RBC (10^6^ *µ*L^−1^)	7.44 ± 0.63	7.14 ± 0.64	7.16 ± 0.52	7.52 ± 0.53
HGB (g dL^−1^)	14.39 ± 1.13	13.94 ± 1.12	14.05 ± 1.02	14.01 ± 1.38
Haematocrit (%)	41.74 ± 3.64	39.43 ± 3.56	39.35 ± 3.12	39.21 ± 3.71
MCV (fL)	56.09 ± 0.76	55.23 ± 1.10	54.93 ± 1.71	53.38 ± 1.10
MCH (pg)	19.36 ± 0.27	19.57 ± 0.54	19.64 ± 0.47	19.09 ± 0.52
MCHC (g dL^−1^)	34.49 ± 0.51	35.39 ± 0.65	35.76 ± 0.48	35.76 ± 0.59
RDW-CV (%)	13.81 ± 1.25	13.89 ± 1.46	13.54 ± 1.38	14.48 ± 1.22
Platelet (10^3^ *µ*L^−1^)	543.50 ± 123.56	736.57 ± 155.47	761.14 ± 148.98	857.25* ± 109.22
MPV (fL)	7.28 ± 0.46	7.09 ± 0.35	7.11 ± 0.42	6.81 ± 0.27

WBC: white blood cell; RBC: red blood cell; HGB: haemoglobin; MCV: mean corpuscular volume; MCH: mean corpuscular Hb; MCHC: mean corpuscular Hb conc; RDW-CV: coefficient variation of red cell distribution width; MPV: mean platelet volume.

*Significantly different from the control group (*P* < 0.05).

**Table 5:** Effect on biochemical parameters after oral administration of lyophilized fruit juice of *Physalis peruviana *L. during subchronic period in male Wistar rats (*n*: 7).

	Male			
	Control	100 mg kg^−1^	1000 mg kg^−1^	5000 mg kg^−1^
ALT (U L^−1^)	78.70 ± 8.26	77.00 ± 15.49	80.00 ± 10.60	86.10 ± 12.32
AST (U L^−1^)	170.84 ± 24.16	168.00 ± 7.79	181.23 ± 112.26	175.86 ± 41.12
ALP (U L^−1^)	137.17 ± 31.35	152.83 ± 16.11	160.33 ± 11.71	183.55 ± 12.97
DBIL (mg dL^−1^)	0.05 ± 0.01	0.05 ± 0.02	0.04 ± 0.01	0.06 ± 0.02
TBIL (mg dL^−1^)	0.08 ± 0.04	0.08 ± 0.03	0.07 ± 0.02	0.07 ± 0.02
Albumin (g dL^−1^)	3.74 ± 0.43	3.75 ± 0.50	3.89 ± 0.29	3.66 ± 0.24
Total protein (g dL^−1^)	6.66 ± 0.34	6.44 ± 0.19	6.74 ± 0.21	6.86 ± 0.49
CRE (mg dL^−1^)	0.32 ± 0.02	0.26 ± 0.05	0.29 ± 0.04	0.32 ± 0.07
Urea (mg dL^−1^)	40.90 ± 5.25	49.10 ± 5.65	50.36 ± 3.45	40.40 ± 7.87
Calcium (mg L^−1^)	10.53 ± 0.30	10.20 ± 0.34	10.70 ± 0.48	10.88 ± 0.60
Chloride (mEq L^−1^)	96.63 ± 3.93	101.04 ± 0.57	100.34 ± 1.01	96.31 ± 3.58
Potassium (mEq L^−1^)	4.78 ± 0.52	4.70 ± 0.46	5.45 ± 1.25	7.42* ± 2.34
Sodium (mEq L^−1^)	144.00 ± 2.14	144.00 ± 1.87	144.43 ± 1.27	144.00 ± 2.45
Glucose (mg dL^−1^)	104.00 ± 16.88	151.14* ± 32.86	151.04* ± 23.62	143.50* ± 8.74
CK-MB (ng mL^−1^)	7.6 ± 0.76	7.10 ± 1.97	9.50 ± 1.06	10.79 ± 2.69
LDH (IU L^−1^)	107.39 ± 12.78	124.31 ± 23.36	132.30 ± 27.90	140.51 ± 21.99
Troponin I (ng mL^−1^)	1.81 ± 0.70	2.64 ± 0.66	2.72 ± 0.69	3.12* ± 0.67
Troponin T (pg mL^−1^)	76.47 ± 25.63	188.48* ± 61.96	165.15* ± 81.32	173.74* ± 51.75

ALT: alanine aminotransferase; AST: aspartate aminotransferase; ALP: alkaline phosphatase; DBIL: direct bilirubin; TBIL: total bilirubin; CRE: creatinine; URE: urea; CK-MB: creatine kinase myocardial band; LDH: lactate dehydrogenase.

*Significantly different from the control group (*P* < 0.05).

**Table 6:** Effect on biochemical parameters after oral administration of lyophilized fruit juice of *Physalis peruviana *L. during subchronic period in female Wistar rats (*n*: 7).

	Female			
	Control	100 mg kg^−1^	1000 mg kg^−1^	5000 mg kg^−1^
ALT (U L^−1^)	72.95 ± 13.08	68.83 ± 10.53	72.00 ± 11.24	69.58 ± 22.40
AST (U L^−1^)	200.29 ± 23.35	199.29 ± 30.86	203.80 ± 22.04	201.57 ± 32.95
ALP (U L^−1^)	132.44 ± 32.82	116.78 ± 21.64	122.20 ± 19.12	129.37 ± 24.64
BILD (mg dL^−1^)	0.04 ± 0.02	0.05 ± 0.01	0.07 ± 0.02	0.05 ± 0.02
BILT (mg dL^−1^)	0.09 ± 0.04	0.12 ± 0.06	0.10 ± 0.02	0.11 ± 0.05
Albumin (g dL^−1^)	3.96 ± 0.34	4.17 ± 0.42	4.13 ± 0.36	3.76 ± 0.51
Total protein (g dL^−1^)	7.13 ± 0.28	7.16 ± 0.37	6.79 ± 0.36	6.83 ± 0.62
CRE (mg dL^−1^)	0.38 ± 0.05	0.34 ± 0.03	0.37 ± 0.05	0.32 ± 0.05
Urea (mg dL^−1^)	48.80 ± 7.60	49.23 ± 9.98	43.74 ± 6.29	44.41 ± 6.04
Calcium (mg L^−1^)	10.68 ± 0.59	10.90 ± 0.29	10.43 ± 0.33	10.16 ± 0.43
Chlorine (mEq L^−1^)	95.89 ± 2.23	97.26 ± 1.55	98.16 ± 1.39	97.89 ± 2.09
Potassium (mEq L^−1^)	4.81 ± 0.29	4.51 ± 0.45	4.52 ± 0.28	4.61 ± 0.38
Sodium (mEq L^−1^)	144.13 ± 1.36	142.86 ± 1.07	142.75 ± 1.28	141.00 ± 1.58
Glucose (mg dL^−1^)	81.95 ± 14.26	120.57 ± 13.72	120.75 ± 14.64	125.50 ± 10.07
CK-MB (ng mL^−1^)	7.23 ± 0.40	10.93 ± 3.17	9.54 ± 1.38	7.71 ± 0.72
LDH (IU L^−1^)	103.32 ± 18.23	109.82 ± 31.86	112.17 ± 36.93	135.4 ± 23.29
Troponin I (ng mL^−1^)	1.60 ± 0.73	3.05* ± 0.41	2.74 ± 0.20	2.72 ± 0.67
Troponin T (pg mL^−1^)	72.31 ± 5.34	91.10 ± 66.09	94.26 ± 36.07	99.00 ± 54.93

ALT: alanine aminotransferase; AST: aspartate aminotransferase; ALP: alkaline phosphatase; BILD: direct bilirubin; BILT: total bilirubin; CRE: creatinine; CK-MB: creatine kinase myocardial band; LDH: lactate dehydrogenase.

*Significantly different from the control group (*P* < 0.05).
